# Oral Health and COVID-19: Increasing the Need for Prevention and Access

**DOI:** 10.5888/pcd17.200266

**Published:** 2020-08-13

**Authors:** Zachary Brian, Jane A. Weintraub

**Affiliations:** 1 North Carolina Oral Health Collaborative, Foundation for Health Leadership and Innovation, Cary, North Carolina; 2 University of North Carolina at Chapel Hill Adams School of Dentistry and Gillings School of Global Public Health, Chapel Hill, North Carolina

## Abstract

Populations disproportionately affected by coronavirus disease 2019 (COVID-19) are also at higher risk for oral diseases and experience oral health and oral health care disparities at higher rates. COVID-19 has led to closure and reduced hours of dental practices except for emergency and urgent services, limiting routine care and prevention. Dental care includes aerosol-generating procedures that can increase viral transmission. The pandemic offers an opportunity for the dental profession to shift more toward nonaerosolizing, prevention-centric approaches to care and away from surgical interventions. Regulatory barrier changes to oral health care access during the pandemic could have a favorable impact if sustained into the future.

**Summary**

**What is already known on this topic?**

Oral health is an important component of health and overall well-being.

**What is added by this report?**

Nonemergency dental care has been curtailed during the coronavirus disease 2019 (COVID-19) pandemic. Reopening dental practices involves unique challenges and provides opportunities to increase focus on prevention and nonaerosol-generating procedures.

**What are the implications for public health practice?**

Vulnerable populations are at high risk for COVID-19 and oral and other chronic diseases, and they also have less access to health care services. Removing policy, regulatory, workforce, and reimbursement barriers and incentivizing prevention would increase access to oral health care and improve population health.

## Introduction

On March 11, 2020, the World Health Organization declared the global spread of coronavirus disease 2019 (COVID-19) a pandemic (1). Severe acute respiratory syndrome coronavirus 2 (SARS-CoV-2) is a new virus with no vaccine or treatment, and the population currently has no immunity. The virus is primarily transmitted by direct or indirect personal contact through airborne respiratory droplets from an infected person (2).

On March 16, 2020, the American Dental Association (ADA), the nation’s largest dental association, recommended that dental practices postpone elective dental procedures until April 6, 2020, and provide emergency-only dental services to help keep patients from burdening hospital emergency departments (3). Because of the rise of infections, this recommendation was updated on April 1, 2020, when the ADA advised offices to remain closed to all but urgent and emergency procedures until April 30 at the earliest. As a result, access to dental care substantially decreased. During the week of March 23, 2020, an ADA Health Policy Institute survey indicated that 76% of dental offices surveyed were closed but seeing emergency patients only, 19% were completely closed, and 5% were open but seeing a lower volume of patients (4).

In addition to the lack of widespread COVID-19 testing, point-of-care testing in dental offices also was not available. Because of the inability to test all patients and the fact that asymptomatic or presymptomatic patients could be infectious, ADA guidance shifted in mid-April 2020 as state and local government policies varied regarding criteria for reopening different types of services, including dental services (5). Questions remain about how soon patients will prioritize and resume nonemergency dental care amid other delayed health care services. The full extent of pandemic-related financial strain and loss of dental insurance is not yet clear and will dramatically affect dental care utilization.

In this commentary, we explain why oral health care should be a public health priority in the response to the pandemic and discuss the aspects of dental care that make it challenging to accomplish this. We will also provide opportunities for improvement, such as focusing more on prevention and nonaerosolizing dental procedures and the means by which to increase access to affordable, more equitable care for vulnerable populations.

## Importance of Oral Health

In 2000, the first and only Surgeon General’s Report on Oral Health (the second is in progress) made clear that oral health is part of overall health and well-being (6). The mouth is indispensable to eating, speaking, smiling, and quality of life. The most prevalent oral conditions are dental caries and periodontal diseases, and they are largely preventable (7). Dental caries is the most common chronic childhood disease and continues into adulthood. Among US adults, 2011–2014 national data indicate that 32.7% had untreated dental caries (8). Furthermore, according to weighted averages from 2009 through 2014, 42% of adults aged 30 or older had periodontitis (9). Oral disease is unevenly distributed in the population by race and ethnicity (Table 1). The progression of oral disease can cause pain, infection, and sepsis, and treatment is expensive. In addition to primary prevention, in early stages the progression can be reversed or arrested with appropriate oral hygiene, fluoride exposure, dental sealants, changes in diet, and other measures.

**Table 1:** Percentage of COVID-19 Hospitalized Cases in COVID-NET Catchment Areas and Prevalence of Dental and Other Chronic Conditions in the United States, by Race/Ethnicity, 2020

Characteristic	% of COVID-19 Hospitalized Cases	COVID-NET Catchment Area for Comparison	% of Periodontitis (Gum Disease)	% of Untreated Dental Caries (Tooth Decay)	% With Diabetes (Physician-Diagnosed and Undiagnosed)	% of Self-Reported Heart Disease
Population	COVID-NET, 14 jurisdictions	COVID-NET, 14 jurisdictions	US dentate adults aged ≥30 y	US dentate adults aged 20–64 y	US adults aged ≥20 y	US adults aged ≥18 y
Period	As of June 20, 2020	As of June 20, 2020	2009–2014	2011–2016	2015–2016	2017
Source	CDC (10)	CDC (10)	NCHS, NHANES (9)	NCHS, NHANES (11)	NCHS, NHANES (12)	NCHS, NHIS (12)
Non-Hispanic White	32.8	58.8	37.0	22.2	13.0	11.5
Non-Hispanic Black	32.6	17.7	56.6	40.2	19.6	9.5
Hispanic	22.0	14.0	a	a	21.5	7.4
Mexican American	a	a	59.7	37.1	a	a
Other Hispanic	a	a	48.5	a	a	a

Abbreviations: CDC, Centers for Disease Control and Prevention; COVID-19, coronavirus disease 2019; COVID-NET, COVID-19–Associated Hospitalization Surveillance Network; NCHS, National Center for Health Statistics; NHANES, National Health and Nutrition Examination Survey; NHIS, National Health Interview Survey.

a Studies vary in definitions used for Hispanic ethnicity.

## Populations With Oral Health and Chronic Disease Disparities: COVID-19 Puts Both at Increased Risk

Populations at higher risk for many chronic diseases are similar to those at higher risk for developing oral diseases. Common risk factors include stress, poor diet, alcohol and tobacco use, substance misuse, behavioral health issues, domestic violence, and poverty. Many of these factors have been heightened during the pandemic. These and other social determinants of health lead to both exacerbation of chronic disease and poor oral health outcomes (13).

Populations vulnerable to COVID-19, including those in low socioeconomic groups, minority groups, older adults, low-literacy individuals, those in rural areas, and the uninsured are also at increased risk for oral disease and associated systemic health problems (14). Minority populations are especially at risk during the COVID-19 pandemic. The Centers for Disease Control and Prevention (CDC) notes that “non-Hispanic blacks, Hispanics, and American Indians and Alaska Natives generally have the poorest oral health of any racial and ethnic groups in the United States,” (15) and these same populations have disproportionately higher incidence of COVID-19–related infection and death (16).

Among those hospitalized with COVID-19, diabetes and cardiovascular disease are 2 of the most prevalent underlying comorbidities, according to the CDC (17). Periodontal disease is associated with diabetes and cardiovascular disease, although causality is difficult to ascertain because of confounding evidence, and few randomized trials or longitudinal studies have been conducted on the effects of treatment (18,19).

Researchers note, “The COVID-19 pandemic has alarming implications for individual and collective health and emotional and social functioning” and that “health care providers have an important role in monitoring psychosocial needs and delivering psychosocial support to their patients” (20). Research suggests a strong association between oral health conditions like erosion, caries, and periodontal disease and mood conditions like stress, anxiety, depression, and loneliness (21). There are other potential connections downstream between COVID-19 and oral health. With the COVID-19 pandemic’s impact on mental health, pandemic-related increases in oral health risk factors, and anticipated declines in per capita dental visits, increasing integrated practice and referrals between dental providers and behavioral health providers will be prudent. Similarly, increased efforts to more effectively integrate dental programs focused on prevention, screening, and risk assessment within primary care, obstetrics and gynecology, and pediatric offices should be pursued to expand access to oral health services for vulnerable populations (22).

## COVID-19 and Oral Health Disparities in Access to Care

Access to oral health care is especially limited for populations at high risk for COVID-19. Patients with symptoms of COVID-19 are advised “to avoid nonemergent dental care” (23). Providers are advised, “if possible, [to] delay dental care until the patient has recovered” (23).

More than 49 million US residents live in areas designated by the Health Resources and Services Administration as Dental Health Professional Shortage Areas (24). This shortage has been compounded by the COVID-19 pandemic, which has resulted in limited preventive dental services in the interest of public health safety. Emergency departments, a less-than-ideal but common treatment destination for those facing oral health care access disparities, have also seen a significant drop in visits for health problems unrelated to COVID-19 (25). School-based oral health programs, such as effective dental sealant programs to prevent dental caries — the only source of preventive oral health care for many children in vulnerable populations — have similarly been suspended because of government-mandated school closures (26). Nationally, children in low-income families and at higher risk of caries are less likely to receive sealants than children in higher-income families, at 39% and 46%, respectively (27).

Access disparities are particularly acute for poor and minority populations. Researchers note that “poor and minority children are substantially less likely to have access to oral health care than their nonpoor and nonminority peers” (14). These populations are also more likely to lack dental insurance. A 2020 report notes, “The oral health care safety net is expected to cover . . . one-third of the US population, notably those who are low-income, uninsured, and/or members of racial/ethnic minority, immigrant, rural, and other underserved groups” (28). Many of these populations, which often rely on Medicaid dental benefits, have seen their access restricted or eliminated by reductions in this vital coverage. In 2020 it was reported that “in response to fiscal challenges, many states have reduced or eliminated Medicaid dental coverage over the past decade, with a concurrent 10% decline in oral health care utilization among low-income adults” (28). Among those in at-risk populations who do have dental benefits under Medicaid, the same report notes there is often “difficulty finding Medicaid-contracted dental providers, because only 20% of dentists nationwide accept Medicaid” (28). We can reasonably anticipate a worsening of these trends as the COVID-19 pandemic takes a large proportion of state budgets.

## COVID-19 and Dental Care: Aerosol-Generating Procedures Create Risk

Dental professionals have been practicing increased infection control and taking universal precautions since the 1980s HIV epidemic (29). Nevertheless, oral health professionals are among those occupations at the highest risk for COVID-19, as reported by *The* *New York Times* (30). Dental care personnel face challenges because of their proximity to infected patients. These patients’ mouths are open and unmasked during treatment, significantly increasing the potential for direct and indirect exposure to infectious materials. The Occupational Safety and Health Administration designates the performance of aerosol-generating procedures on known or suspected COVID-19 patients as “very high risk” (31). Shortages of personal protective equipment (PPE) and the use of instruments and equipment that generate aerosols containing oral and respiratory fluids only compound the risk (23). Two of the highest aerosol-creating procedures involve inventions that have been considered major advances in dental practice, because they are faster and less painful for the patient: the high-speed handpiece with its water spray coolant and the ultrasonic scaler used by hygienists to remove hard deposits on teeth (32). These dental procedures have become problematic during the pandemic, providing an opportunity to shift to nonaerosolizing procedures and a greater focus on prevention (23,33).

## Going Forward: Opportunities

### Focus on prevention and promote nonaerosol-generating dental procedures

Prevention is a cornerstone of public health. The COVID-19 pandemic presents an opportunity for the dental profession to shift from an approach focused on surgical intervention to one emphasizing prevention. Embracing nonsurgical, nonaerosolizing caries prevention and management will be critical in this endeavor. The profession has always supported community water fluoridation, and dental hygienists are considered prevention experts (34,35). However, the dental compensation model is based on providing expensive, restorative procedures that are financially out of reach for many people.

Guidelines have been developed to shift the dental care paradigm to a more preventive focus (36–40). Strategies include reduction in common risk factors such as tobacco and alcohol use, promotion of a healthy diet low in sugars, community water fluoridation, topical fluorides, and promotion of oral health in community settings. These oral health messages and interventions should be integrated into medical sites such as primary care and pediatric offices. Prevention and nonsurgical caries management include many options. Evidence-based materials include dental resin sealants, glass ionomers as sealants or as part of atraumatic restorative treatment performed with hand instruments, silver diamine fluoride, sodium fluoride varnish, and other self-applied and professionally applied topical fluorides (40–42). These materials can be applied without generating aerosols, reducing the risk of viral transmission. These methods present a major opportunity to expand access to preventive and restorative care for vulnerable populations, particularly when combined with policy changes increasing hygienists’ scope of practice, sustainable payment reform, and changes in the education of oral health professionals.

Providers and payers together have a responsibility to shift toward preventive care, particularly as COVID-19 threatens to increase disparities in oral health care access for the United States’ most vulnerable populations. Before the pandemic, Birch et al noted that a review of provider and payer practices made clear that “further work was required on both the provider and payer side to ensure that evidence-based prevention was both implemented properly but also reimbursed sufficiently” (43). As health care compensation moves toward value-based care and a focus on health outcomes, prevention and maintaining oral health and sound tooth structure will shift reimbursement away from the current expensive model of reimbursement for restoration of tooth structure and function (44). In particular, reimbursement policies, which traditionally have incentivized surgical, high-end restorative procedures like crowns and multisurface fillings, must be revisited to prioritize preventive and nonsurgical, nonaerosolizing treatments and make them more financially sustainable.

### Improve communication

Communications concerning patient and provider safety are critical (45). Surveillance and monitoring are needed to confirm whether transmission of COVID-19 occurs in the dental office. According to CDC (27), “There are currently no data available to assess the risk of SARS-CoV-2 transmission during dental practice.” The availability of PPE for dental care should be monitored, and the effectiveness of various types of PPE should be determined. Many oral health care providers are anxious about returning to work, and many patients may be hesitant to enter a dental office. Communication and clarity are critical, especially with low-literacy populations. Messaging should include the importance of maintaining good oral health and its role in overall health.

### Protect and enhance Medicaid reimbursement

Dental coverage under Medicaid is mandated for children, but state Medicaid programs’ approaches to oral health services for adults vary significantly, especially in terms of the comprehensive nature of such services (Figure). Only 19 states have “extensive” Medicaid dental benefits for adults (46). Among US adults aged 19 to 64, only 7.4% have Medicaid dental benefits and, alarmingly, 33.6% have no dental insurance benefits (47). The fiscal solvency of dental safety-net clinics will thus remain critical to serving at-risk populations during and after the pandemic. These sites will be needed more than ever, as delayed and postponed treatment increases need for more extensive and urgent care.

**Figure:**
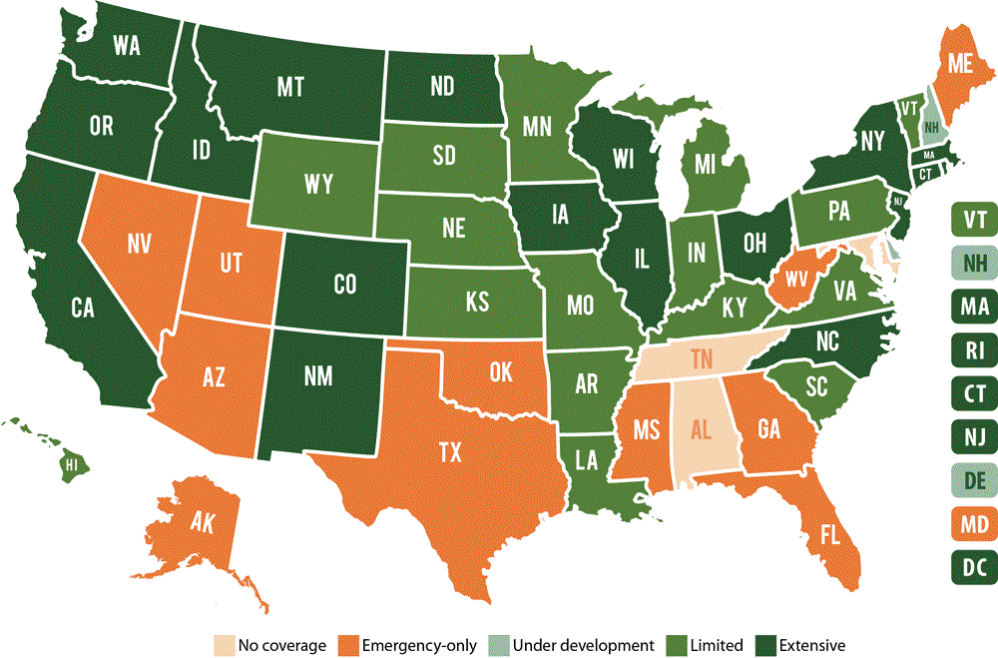
Extent of Medicaid adult dental benefits, by state. Source: Center for Health Care Strategies (46).

It is widely documented that during economic downturns, Medicaid enrollment increases (48). With unemployment increasing at an unprecedented rate, we can reasonably anticipate the same effect in this pandemic. During times of state budget cuts, dental Medicaid coverage is often at risk (49). In the immediate aftermath of the Great Recession during state fiscal years 2010 through 2012, 19 states reported restrictions in Medicaid adult dental benefits (50). Amidst the pandemic, many states have modified public payment policies to meet the demand of their most vulnerable residents, and it will be important that advocacy efforts secure continuity of these provisional changes. However, given current circumstances, it is imperative that policy makers consider expanding adult dental benefits under Medicaid rather than reducing them. Access disparities will likely increase without expansion of dental benefits under Medicaid.

### Ease dental workforce restrictions

Guidance for dental practice during COVID-19 continues to evolve, and regulations vary by state (51). As dental care resumes, it is critical that workforce policies and licensure scope are evaluated to address workforce utilization bottlenecks to respond to communities’ needs more effectively and efficiently.

As of 2019, 11 states did not allow for some form of direct access to preventive oral health services by a dental team member outside of the dentist’s supervision (52). In these states, a dentist must perform an examination before delivery of preventive care by a hygienist. Easing scope of practice and workforce restrictions would increase access to care. Increasing opportunities for dental team members like dental therapists, community dental health coordinators, and expanded function dental assistants — all currently in limited supply and restricted by dental practice acts in many states — would help bring needed, more affordable services to underserved communities.

### Advance teledentistry to address access gaps

The COVID-19 pandemic has thrust alternative modalities such as teledentistry to the forefront of policy considerations (53). Teledentistry supports the delivery of oral health services through electronic communication means, connecting providers and patients without usual time and space constraints. Teledentistry’s unique ability to connect disadvantaged, primarily rural communities and the homebound with dental providers (54) makes this method particularly well-suited to address lack of access during and after the pandemic.

Teledentistry can be used for education, consultation, and triage, allowing providers to advise patients whether their dental concerns constitute a need for urgent or emergency care, whether a condition could be temporarily alleviated at home, or whether treatment could be postponed. When many dental offices are closed and people are largely staying at home, communication and information via teledentistry can help lessen the burden of people seeking dental care at overwhelmed emergency departments and urgent dental care settings. In more usual circumstances, teledentistry can also be used to facilitate access to preventive services and oral health education when members of the dental team can provide such services in community settings, such as schools, without onsite dentist supervision.

Before COVID-19, many states inhibited use of teledentistry through legislative barriers and limited public and private insurance reimbursement. Compared with dentistry, many medical and behavioral health providers have less restrictive regulations and insurance reimbursement policies concerning telehealth. A *Washington Post* report (55) was clear: “Telemedicine was largely ready for the influx.” Teledentistry, on the other hand, was forced to play catch-up (56). Emergency reimbursement changes prompted by COVID-19 have brought relief, but post-pandemic, we recommend that legislators, regulatory authorities, and third-party payers consider making permanent the temporary modifications to teledentistry policies to support increased access.

## Implications for Public Health Practice: Dental Public Health’s Roles

Health inequities are avoidable and unjust. Although SARS-Cov-2 has infected people worldwide, it has disproportionately affected those who are most disadvantaged. In the United States, people without good access to health care, healthy food, and a safe environment; with underlying health conditions; who live in crowded conditions; or who have become unemployed and homeless are especially vulnerable and at increased exposure to the virus. It is time to recognize the social determinants of health and rectify unjust conditions, systemic inequality, and racism.

Oral health disparities and inequities are part of the larger, cultural picture. There has been a tendency to blame the victim. Mary Otto, health journalist and author of the groundbreaking book *Teeth* (57), stated, “We see tooth decay through a moral lens, almost. We judge people who have oral disease as moral failures, rather than people who are suffering from a disease” (58).

It is perhaps not hyperbole to describe pandemic-related circumstances as creating a “perfect storm” in oral health care in the United States. Risk factors are elevated, access for the most vulnerable is limited, safety concerns are heightened, and the economy presents substantial challenges for patients and providers alike. The effects of COVID-19 are particularly acute for vulnerable populations, and the crisis has made evident the challenges and opportunities for oral health care in the United States. In such a time, oral health care providers and advocates must clearly communicate the importance of oral health to overall health, indicate the steps being taken to ensure patient and provider safety, and promote prevention and nonaerosolizing procedures (Table 2). Oral health should be included in policy considerations, continued research, monitoring, surveillance, and other aspects of health. Advocacy is crucial to make permanent the temporary regulatory changes being implemented to address the immediate crisis, ensure access to oral health care, address disparities and inequities, and improve population health. 

**Table 2:** Implications of COVID-19 for Oral Health in the United States, 2020

Core Functions of Public Health	Public Health Concerns	Future Opportunities
**Assurance**	Limited access to dental care compounded by COVID-19; aerosol-generating dental procedures increase risk of transmission	Promote prevention and use of nonaerosol-generating dental procedures; advance teledentistry training and reimbursement and other efforts to reach patients outside of the dental setting
	Regulations in some states limit dental hygienists’ and other dental team members’ ability to provide care in settings outside of the dental office	Modify state dental practice acts and other regulations for dental workforce reform and to increase access to prevention
	Lack of integration between oral health and the rest of the health care system	Increase integration between oral health care and primary care (ie, locations serving patients who are pregnant, have diabetes or cardiovascular disease)
**Assessment**	Lack of timely national oral health data and coordinated state and local information	Monitor oral health conditions as a result of delayed dental care during pandemic; include oral health metrics in health care quality measures
	Lack of information about health and safety of dental health care personnel during COVID-19; limited availability of PPE and COVID-19 testing for dental practices	Monitor dental workforce health and safety; increase availability of PPE and COVID-19 tests for dental care settings
	Evidence needed to determine most cost-effective PPE or PPE combinations and other measures to prevent SARS-CoV-2 in dental settings	Further testing of specific PPE and PPE combinations and other measures to protect patient and provider health in dental settings
**Policy Development**	Potential public and provider unease about seeking and providing dental care during pandemic	Provide clear communication about how to safely obtain and provide dental care during the pandemic
	Oral health not prioritized	Educate about importance of oral health and its relation to the health of the rest of the body; provide parity with health care policies (ie, Medicaid, Medicare)
	Varied state-level adult dental Medicaid benefits	Advocate for sustained dental Medicaid funding and expansion to close coverage gaps
	Reimbursement models incentivize surgical, high-end restorative dental procedures	Modify reimbursement to provide incentives for prevention, maintaining health, teledentistry

Abbreviations: COVID-19, coronavirus disease 2019; PPE, personal protective equipment; SARS-CoV-2, severe acute respiratory syndrome coronavirus 2.
