# Erratum, Vol. 17, August 13 Release

**DOI:** 10.5888/pcd17.200266e

**Published:** 2020-08-27

In the article, Oral Health and COVID-19: Increasing the Need for Prevention and Access, the degree designations were omitted for Jane A. Weintraub, DDS, MPH. The author byline was corrected on August 14, 2020, and the article appears online at https://www.cdc.gov/pcd/issues/2020/20_0266.htm. We regret any confusion or inconvenience this error may have caused.

